# An evidence-informed occupational health curriculum for pre-deployment preparation of aquanauts in underwater habitats: a scenario-based framework development study

**DOI:** 10.3389/fpubh.2026.1962851

**Published:** 2026-09-25

**Authors:** Inho Lee, Jisuk Yun, Dongwoo Kim, Hyeon-Ah Lee, Young-Sun Min, Hee-Dong Kim, Hyung Jun Moon, Gi Woon Kim, Hwa-Young Lee

**Affiliations:** 1 Department of Occupational and Environmental Medicine, Soonchunhyang University Cheonan Hospital, Cheonan, Republic of Korea; 2 Department of Occupational and Environmental Medicine, Chungbuk National University Hospital, Cheongju, Republic of Korea; 3 Department of Occupational and Environmental Medicine, Soonchunhyang University Seoul Hospital, Seoul, Republic of Korea; 4 Department of Psychiatry, Soonchunhyang University Cheonan Hospital, Cheonan, Republic of Korea; 5 Division of Cardiology, Department of Internal Medicine, Soonchunhyang University Cheonan Hospital, Cheonan, Republic of Korea; 6 Department of Emergency Medicine, Ajou University Medical Center, Suwon, Republic of Korea; 7 Department of Emergency Medicine, Soonchunhyang University Bucheon Hospital, Bucheon, Republic of Korea

**Keywords:** aquanaut, underwater habitat, occupational health and safety, pre-deployment training, curriculum development, isolated and confined environments, diving medicine

## Abstract

**Background:**

Underwater habitats represent emerging extreme workplaces in which pressure exposure, immersion, confined living, external underwater tasks, and delayed access to definitive care create combined occupational health and safety demands. Candidate selection establishes baseline suitability. It cannot, on its own, specify which preventive and response capabilities should be taught, practiced, and documented between selection and deployment.

**Methods:**

We developed a scenario-based, evidence-informed occupational health curriculum for the pre-deployment preparation of non-medical research personnel. The design case was deliberately narrow. It placed three to six crew members in a 15-m habitat for 14 days, with topside telemedical support and limited immediate specialist care. Peer-reviewed studies and official manuals were mapped to mission phases, occupational and operational risk events, expected actor actions, and feasible non-medical performance. No Delphi round or validation study was undertaken. Internal project documents only defined the scenario.

**Results:**

The occupational health curriculum specification contains four domains, ten modules, and 28 observable learning outcomes. Emergency preparedness, physical self-maintenance, psychological and team functioning, and underwater operational safety are separated by immediate performance purpose. Together, the domains connect worker protection across routine prevention, acute response, psychosocial risk management, and task-specific safety. Three modules carry the psychological and team content. Sleep and circadian management, emotional self-regulation, and crew communication with conflict management are each specified on their own.

**Conclusion:**

This article offers a preliminary occupational health curriculum for pre-deployment preparation that underwater habitat programs can adapt. It is evidence-informed and unvalidated. The framework addresses worker protection within a bounded underwater workplace while preserving the authority of diving, clinical, and mission-governance systems. Independent stakeholder review, instructor usability testing, pilot training, and analog-mission observation remain necessary before the framework informs any operational readiness decision.

## Introduction

Underwater habitats are emerging as extreme workplaces for long-duration marine research, environmental monitoring, infrastructure work, and exploration. Workers in these settings occupy a hybrid occupational role: resident, team member, and task performer inside a pressurized structure. Their preparation therefore involves more than diving technique or medical selection alone. A recent Korean selection model sets out psychological evaluation criteria and the procedures around them, applied before a candidate enters such a habitat (1). Selection is necessary. It is not a curriculum.

The Aquarius habitat and the National Aeronautics and Space Administration Extreme Environment Mission Operations program show why a habitat mission amounts to more than a series of dives. Multiday saturation, habitat living, external tasks, limited resources, and coordinated crew work all occur inside one operational system (2, 3). Underwater habitation also shares central features with isolated, confined, and extreme (ICE) environments: reduced privacy, restricted egress, constrained communication, and sustained dependence on a small team (4, 5). That combination turns preparation into a public and occupational health question. The technical prerequisites are only part of it.

Physiological and environmental hazards remain central. Immersion redistributes blood centrally and alters respiratory and cardiovascular loading, while pressure change introduces decompression illness, pulmonary barotrauma, oxygen toxicity, nitrogen narcosis, and carbon dioxide accumulation (6–9). Heat loss, awkward working postures, repeated equipment handling, and variable task pacing all feed into fatigue and functional performance. The prevention target follows from this. A worker must recognize a change, take a safe first action, communicate accurately, and escalate within the limits of the assigned role.

Psychological and team factors carry equal weight. Studies of spaceflight and analog missions identify role clarity, cooperation, communication, stress regulation, sleep, and conflict management as recurrent determinants of small-crew functioning (10–12). Sleep restriction has been documented before, during, and after spaceflight, and earlier astronaut programs deliberately included training in stress coping, interpersonal functioning, and crisis response (13–16). None of this makes a habitat equivalent to a spacecraft. What it provides is a defensible analogue for designing preventive learning in a similarly constrained team environment.

Medical access adds a further layer of risk. Telemedicine can support assessment, instruction, and escalation where local resources are thin, provided that the transmitted information is complete and a local care pathway exists (17). Resuscitation guidance emphasizes early recognition, prompt basic life support (BLS), defibrillation when indicated, and regular skills practice (18–20). Diving medicine guidance gives similar priority to prompt first aid, oxygen where indicated, clinical communication, and coordination with hyperbaric or emergency services once decompression illness is suspected (21, 22). Access, communication, and patient movement complicate even familiar emergencies inside confined spaces (23).

Occupational health provides the integrating perspective. Saturation divers describe pressures tied to uncertainty, monotony, limited privacy, and the sustained need to adapt inside a small living and working space (24). Scientific and naval diving standards emphasize pre-dive checks, briefings, buddy systems, equipment control, and emergency procedures (25, 26). Submarine platforms supply further evidence that medical evacuation and psychological hygiene bear directly on mission continuity (27, 28). In an underwater habitat, occupational health spans hazard recognition, fatigue and ergonomic risk control, routine health maintenance, psychosocial risk management, emergency escalation, and coordination with diving operations. The curriculum therefore addresses worker protection within a defined work system rather than general aquanaut education. It substitutes for no professional diver qualification, statutory training, or clinical care.

Existing training frameworks sometimes combine development with in-house ratings labeled as validation, which can obscure the transition from evidence to content. Here, development is reported separately. The contribution is a traceable account of how one bounded mission scenario was converted into modules and learning outcomes; external review and empirical testing are reserved for subsequent work.

The aim was to specify an evidence-informed occupational health curriculum for the pre-deployment preparation of non-medical personnel who may live and work in a 15-m underwater habitat for roughly 2 weeks. The educational target remained deliberately narrow. Five objectives followed. We defined mission boundary conditions, mapped occupational and operational risk events to feasible worker actions, derived the domain and module architecture, listed every learning outcome, and separated formative implementation evidence from any later readiness decision.

A curriculum earns its place at the boundary between selection and deployment because it makes organizational expectations discussable. Screening describes a person’s baseline status at one point in time. Without a linked educational process, it cannot show whether that person has rehearsed a structured handoff, can recognize a loss of environmental control, has practiced a task-abort signal, or knows how to raise a sleep or mood problem. These behaviors are trainable and context-dependent. A program should specify them before claiming to observe them.

The intended audience extends beyond any single future mission. Occupational health clinicians, diving medicine advisors, emergency instructors, operational supervisors, and instructional designers all need a common language for a constrained underwater workplace, and the framework aims to supply one. The occupational health perspective links worker-level prevention and health support to operational controls governed by diving, emergency, and mission-management systems. It creates no new medical standard of care. Neither does it define an individual’s fitness to dive. Its purpose is to render the pre-deployment educational component explicit enough to be adapted, taught, audited, and eventually studied.

## Methods

### Study design and positioning

This was a scenario-based framework-development study. Its product is a preliminary occupational health educational specification; no clinical, behavioral, or safety intervention was tested. The occupational health perspective served as the integrating frame for translating workplace hazards and health-support needs into trainable, role-bounded worker capabilities. Guidance for complex interventions informed the development process; that guidance emphasizes context, uncertainty, implementation, and later evaluation rather than treating development itself as proof of effectiveness (29, 30). Traceability principles drawn from framework analysis and from intervention-description guidance were also adopted (31, 32).

Five analytic steps were used: (1) define the intended use and mission boundary; (2) identify evidence sources relevant to that boundary; (3) extract risk-action statements; (4) group statements into separable capability functions; and (5) describe modules, learning outcomes, delivery methods, and candidate implementation evidence. The sequence was explicit but iterative. Table 1 records each step with its decision rule and reported output. Development began with eight learning-need clusters (C1–C8) and ended in the four-domain, 10-module architecture. Figure 1 summarizes the final pathway from the mission design case to evidence mapping, risk-action statements, curriculum structure, formative observation, and future validation.

**Table 1:** Transparent development pathway and outputs.

Step	Input or analytic question	Decision rule	Output reported in this manuscript
1. Define use case	Who will be trained, in what setting, and for what mission?	Fix depth, duration, crew role, work tasks, and medical-access assumptions before selecting curriculum content.	Standardized scenario and exclusions (Table 2).
2. Map evidence	Which published and official sources identify risks or worker actions relevant to the scenario?	Retain sources that address a scenario-relevant risk and an actionable prevention, response, communication, or control.	Search concepts, explicit syntax, repositories, and source inventory (Methods; Supplementary Tables S2, S3).
3. Extract risk-action statements	What could go wrong, and what can a non-medical crew member reasonably do?	Record mission phase, hazard, consequence, expected action, and escalation condition.	Risk-capability matrix summarized by modules and outcomes (Table 3).
4. Form capability functions	Which actions require distinct teaching, privacy safeguards, or observation methods?	Separate functions when their immediate performance purpose and observable evidence differ.	Four domains and ten modules; sleep, stress, and team conflict separated.
5. Specify implementation	How could the content be taught and observed without claiming validation?	State recipient, content, delivery, observation context, and candidate formative evidence; treat duration as adaptable and omit readiness thresholds.	Illustrative 40-h example (Table 4) and separate formative/health-support blueprint (Table 5).

**Figure 1:**
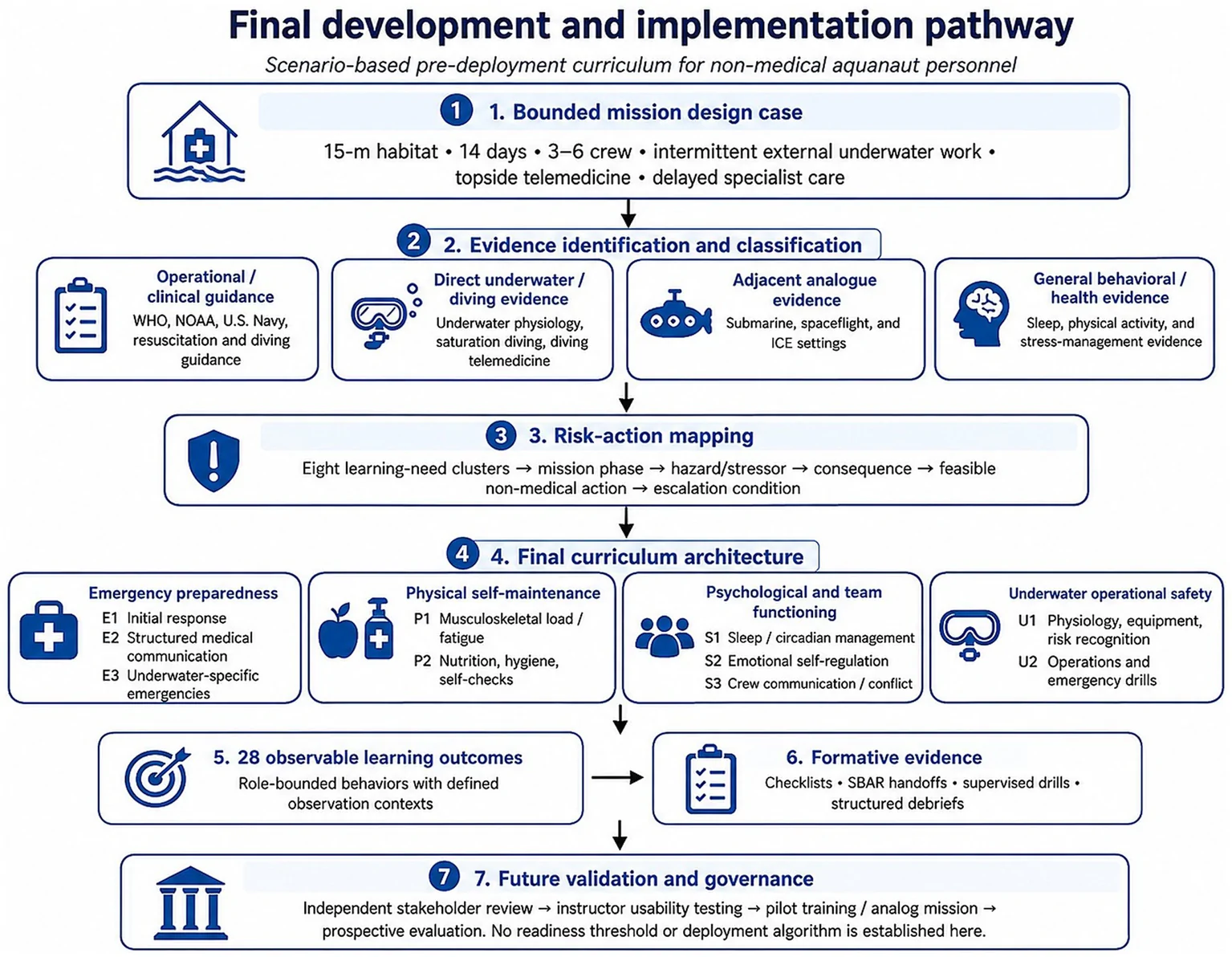
Final development and implementation pathway for the proposed occupational health pre-deployment curriculum. A bounded underwater-habitat workplace design case guides evidence identification and risk-action mapping. Evidence is classified as authoritative operational/clinical guidance, direct underwater/diving evidence, adjacent analogue evidence, or general behavioral/health evidence. Eight learning-need clusters are resolved into four domains, ten modules, and 28 observable outcomes that organize worker protection across routine prevention, health support, acute response, and task-specific safety. Formative observations support feedback and remediation; readiness decisions remain outside the present framework and require independent governance and prospective validation.

The development process was iterative. A risk-action statement could prompt revision of scenario wording; a proposed outcome could expose an intended actor being asked to perform a professional task; and a module could be split when its learning mechanism proved heterogeneous. Alternative configurations were therefore considered. The final structure was retained only when each module met the stated boundary rule.

### Mission scenario and intended users

The framework was bounded to one standardized design case and is not a universal underwater curriculum. The 15-m depth, 14-day stay, crew size of three to six, intermittent external work, telemedical support, and delayed specialist care were taken from internal planning assumptions for the underlying underwater-habitat research project. They define the case. These values were not selected as generic aquanaut standards. External underwater tasks were assumed only for personnel who already held the diving qualification, medical clearance, and site authorization required by the applicable jurisdiction and operating organization. The curriculum does not provide entry-level or statutory diver certification. Table 2 states the remaining operating assumptions and exclusions.

**Table 2:** Standardized mission scenario and boundary conditions.

Parameter	Assumption in this framework	Implication for curriculum scope
Habitat exposure	15-m underwater habitat	Models a shallow, planned habitat setting; it does not model deeper or prolonged saturation exposure.
Residence duration	14 continuous days	Requires preparation for routine self-management, sleep disruption, and sustained teamwork.
Crew	Three to six non-medical research personnel	Defines a peer-dependent crew with limited on-site clinical capacity.
Work profile	Habitation, maintenance, intermittent external underwater work, and emergency response	Combines daily preventive practices with operational and emergency actions.
Medical support	Topside telemedical consultation may be available	Requires structured information transfer and predefined escalation links.
Definitive care	Immediate specialist care may be limited or delayed	Supports first-response training within a non-medical scope; it does not justify independent diagnosis or advanced procedures.
Operational controls	Local diving rules, equipment specifications, emergency plans, medical governance, and credentialing remain site-specific	The framework must be adapted to the actual operation before use.
Scenario provenance	15-m and 14-day values taken from internal planning assumptions for the underlying project	Treats the scenario as a bounded design case, not a generic aquanaut standard.
Prerequisite diving status	External underwater tasks are limited to personnel already holding the qualification, medical clearance, and authorization required locally	U1/U2 supplement existing certification and cannot create or replace professional diving competence.
Excluded use	Commercial, military, medically staffed, deeper, or longer-duration missions	No claim of direct applicability to settings outside the stated boundary.

### Evidence identification and source selection

A structured evidence-mapping exercise drew on PubMed and the public document repositories of the World Health Organization (WHO), the National Oceanic and Atmospheric Administration (NOAA), the United States Navy, and the National Aeronautics and Space Administration. The last evidence update was May 4, 2026. Search concepts paired underwater habitat or saturation exposure with diving medicine, decompression illness, telemedicine, isolated and confined environments, crew mental health, sleep, physical conditioning, emergency response, and diving safety. For reproducibility, Supplementary Table S2 reports explicit PubMed syntax corresponding to these concept blocks, repository-specific query terms, search dates, and selection logic.

The search sought established hazards, training-relevant actions, and implementation considerations. Records were retained when they addressed: (a) an underwater, diving, pressure, submarine, or ICE analogue setting; (b) a preventable or manageable health and safety risk; (c) a worker action, skill, or organizational control able to inform training; and (d) a population or task relevant to the defined scenario. Sources were excluded when they offered no scenario-relevant risk-action link or required specialist procedures outside the intended non-medical role.

Scoping methods support transparent mapping where the aim is to clarify an emerging field rather than estimate an effect (33). This exercise was not a systematic scoping review. The search was purposive, study-quality appraisal and independent duplicate screening were omitted, no formal screening count was calculated, and no prespecified saturation rule was used. The explicit search syntax in Supplementary Table S2 is therefore a reproducibility aid for the reported concept blocks, not a retrospectively reconstructed PRISMA-style screening log.

Extraction distinguished four evidence roles. Authoritative operational or clinical guidance specified procedures and role limits. Direct underwater or diving evidence described setting-specific hazards and actions. Adjacent analogue evidence from submarine, spaceflight, or ICE settings informed contextual features such as sleep, team coordination, and psychological support. General behavioral or health-intervention evidence informed transferable self-management strategies. Supplementary Tables S1, S3 make these evidence roles visible.

No numerical quality score weighted the sources. Operational and clinical guidance took priority for procedures, role boundaries, and escalation. Direct underwater or diving evidence took priority over analogue evidence for setting-specific hazards. Analogue or general evidence never overrode site-specific diving requirements. Where evidence supported only a broad preventive principle, the curriculum calls for local operationalization. Uncertainty was retained rather than converted into a universal procedure.

The evidence matrix carried a provenance note against every outcome: the source family identifying the risk, the reason the behavior remained within a non-medical scope, and the module in which it could be practiced. Supplementary Table S1 provides the outcome-level audit, and Supplementary Table S3 inventories every retained source with its evidence role and primary curricular use. No statistical association was estimated from the matrix. Its purpose was traceability. Any proposed outcome lacking a risk, feasible action, or defensible evidence source was revised or dropped.

### Risk-capability mapping and curriculum rules

For each retained source, the development team recorded the operational setting, mission phase, risk event or stressor, likely consequence, expected non-medical actor action, and any performance cue or escalation condition. Four mission phases were coded: routine habitation, task preparation, external underwater operations, and emergency or evacuation. Expected actions were coded as recognition, self-maintenance, communication and escalation, or operational control. IL conducted the initial extraction and coding. Domain-specific review was then undertaken by H-AL and H-YL for psychological and team content, Y-SM and H-DK for occupational health and physical content, and HJM and GWK for emergency and operational-safety content; JY and DK reviewed the integrated framework. Coding was collaborative, not independently duplicated. Disagreements were resolved by discussion against the predefined retention and module-boundary rules, and no inter-rater reliability statistic was calculated.

One rule governed module boundaries. The matrix was examined for functions requiring different learning methods or different performance evidence, and a capability moved into its own module when it had a distinct immediate purpose and could be observed independently during training. This rule separated sleep and circadian management from emotional self-regulation, and both from crew communication and conflict management. It also separated emergency stabilization from structured medical communication and evacuation linkage. Competing configurations were reconsidered whenever these distinctions were unclear.

A learning outcome was retained on three conditions. It had to be traceable to a defined risk-action statement, lie within a non-medical crew member’s scope, and rest on at least one official operational or clinical guideline or on convergent peer-reviewed sources. Outcomes used observable verbs and a defined context. Observation contexts include accredited skills instruction, role-play, locally approved checklists, and supervised drills. These are performance conditions. They are not universal pass/fail standards; thresholds remain the responsibility of accredited programs, local governance, and future validation.

The matrix also separated an individual task from a crew task. Monitoring fatigue, preparing a sleep plan, and recording a new symptom sit on the individual side. Closed-loop confirmation, a structured handoff, a response to changing environmental conditions, and a task-abort decision sit on the crew side. The separation carries practical weight. A worker can understand a procedure and still fail to enact it under team pressure, and a group can look coordinated while one member’s health concern goes unheard.

Items were excluded when they demanded clinical diagnosis, invasive treatment, specialized rescue authority, or equipment-specific proficiency that could not be safely generalized. Recognition outcomes therefore refer to predefined warning signs and activation criteria rather than diagnostic differentiation. Such specialist items may belong in a local professional curriculum. They are outside this one. The exclusion rule keeps the framework anchored to prevention, trained first actions, and escalation.

Some outcomes stand in a prerequisite relationship. A participant may need basic knowledge of pressure-related risk before an underwater emergency scenario becomes meaningful, and a crew may need a shared handoff structure before remote consultation can be rehearsed. The framework therefore recommends a sequence running from orientation and individual skills to crew communication and integrated drills. The sequence reflects pedagogy alone. It should be adjusted when local requirements impose a different order.

### Curriculum specification and implementation evidence

Replication was the organizing concern. The curriculum was specified through intended recipients, rationale, content, mode of delivery, materials or setting, facilitator requirements, and candidate evidence of learning. This follows the logic of the TIDieR framework while stopping short of presenting the curriculum as an evaluated intervention (32). Duration remains adaptable. Table 3 carries the complete learning-outcome set, while Table 4 gives one illustrative 40-h implementation example rather than a recommended minimum or validated training dose.

**Table 3:** Complete set of 28 learning outcomes in the occupational health pre-deployment curriculum.

Code	Module	Observable learning outcome	Primary risk-capability anchor
E1-1	E1	Recognize predefined warning signs requiring immediate trained response or escalation, including unresponsiveness with abnormal breathing, airway obstruction, major bleeding, signs of poor perfusion, altered consciousness, and rapidly worsening respiratory symptoms.	Red-flag recognition and activation
E1-2	E1	Demonstrate trained basic life support, AED use, airway-obstruction response, and bleeding-control steps within the scope of the local training program.	Initial stabilization
E1-3	E1	State role limits, activate emergency support, and preserve scene safety when a condition exceeds non-medical crew capability.	Safe escalation
E2-1	E2	Deliver a structured SBAR-style account of the event, relevant history, observations, actions already taken, and requested support.	Structured communication
E2-2	E2	Obtain, record, and transmit available vital signs and operational context with units, time points, and observed change.	Information quality
E2-3	E2	Use the locally approved telemedical-contact and medical-kit procedure while documenting the advice received and actions agreed.	Remote-support linkage
E3-1	E3	Recognize predefined warning signs or exposure histories that trigger trained first actions and escalation for possible decompression illness, barotrauma, fire or smoke exposure, carbon dioxide concern, or trauma, without requiring diagnostic differentiation.	Habitat- and pressure-specific emergency recognition
E3-2	E3	Initiate trained first actions, crew protection, and evacuation preparation for the relevant scenario while avoiding actions outside the assigned role.	Scenario-based response
E3-3	E3	Use local decision aids to organize a handoff, evacuation request, and transport preparation.	Evacuation linkage
P1-1	P1	Demonstrate a locally adapted warm-up, movement, and equipment-handling technique for anticipated maintenance or underwater tasks.	Musculoskeletal load reduction
P1-2	P1	Identify personal and team indicators of musculoskeletal strain, fatigue, reduced concentration, or unsafe work pacing.	Fatigue recognition
P1-3	P1	Apply work pacing, recovery, task rotation, and stop/report rules specified by the local operation.	Fatigue control and escalation
P2-1	P2	Prepare and follow an individualized plan for hydration, meals, permitted medicines, hygiene, and daily self-checks.	Routine health maintenance
P2-2	P2	Apply basic skin, wound, gastrointestinal, and infection-prevention measures relevant to the habitat environment.	Preventive self-care
P2-3	P2	Record a relevant new symptom or self-check change and communicate it through the established pathway.	Early reporting
S1-1	S1	Identify schedule, light, noise, stimulation, and behavior factors that may disrupt sleep or circadian timing in the habitat.	Sleep/circadian risk recognition
S1-2	S1	Create a practical sleep opportunity and fatigue-management plan that fits the mission schedule and local controls.	Sleep planning
S1-3	S1	Maintain a brief sleep/fatigue record and recognize when a change requires discussion or clinical escalation.	Monitoring and escalation
S2-1	S2	Use a structured method to recognize stress, anxiety, irritability, or panic-like activation during a task or routine period.	Emotional-state recognition
S2-2	S2	Practice an agreed self-regulation strategy, such as paced breathing, brief relaxation, attention reset, or cognitive reframing.	Self-regulation
S2-3	S2	Prepare a confidential help-seeking and escalation plan for acute psychological distress.	Psychological safety
S3-1	S3	Use routine crew check-ins, role clarification, and closed-loop communication during planned work and emerging problems.	Team coordination
S3-2	S3	Apply a structured, respectful process for raising a concern, clarifying an operational disagreement, and seeking facilitated resolution.	Conflict management
S3-3	S3	Recognize when peer support is insufficient and activate the defined supervisor, medical, or mental-health escalation route.	Peer-support boundary
U1-1	U1	Explain, at the level required for the assigned role, how pressure change, gases, buoyancy, and equipment faults can create risk.	Diving physiology and hazard recognition
U1-2	U1	For personnel already qualified and authorized for external underwater work, complete locally required pre-dive equipment, communication, and buddy checks before an assigned task.	Pre-task operational control
U2-1	U2	For personnel already qualified and authorized for external underwater work, demonstrate safe entry/exit, communication, line or tether management, and task-abort signaling in a supervised drill.	Underwater work procedure
U2-2	U2	For personnel already qualified and authorized for the assigned diving role, participate in a supervised response to a lost-diver, entanglement, equipment-failure, or casualty-extraction scenario within local role limits.	Emergency operational drill

**Table 4:** Illustrative 40-h implementation example for the occupational health pre-deployment curriculum and candidate evidence of learning.

Code	Module	Delivery approach	Time	Candidate formative evidence
E1	Initial response to life-threatening events	Accredited BLS/AED instruction, skills stations, brief scenario practice	4 h	Instructor checklist and feedback on trained basic response steps
E2	Structured medical communication and telemedicine	Case-based SBAR practice, role-play, medical-kit orientation	4 h	Documented structured handoff and information-transmission feedback
E3	Underwater-specific emergencies and evacuation linkage	Scenario simulation, local emergency-plan walk-through, debriefing	4 h	Team rehearsal record and debriefed escalation sequence
P1	Musculoskeletal load and fatigue management	Practical workshop, task pacing, movement and equipment-handling practice	4 h	Observed safe movement/pacing plan and reflection on stop/report rules
P2	Nutrition, hydration, hygiene, medication, and self-checks	Short teaching units, checklist rehearsal, individualized planning	4 h	Completed daily self-management plan and reporting pathway
S1	Sleep and circadian management	Interactive workshop, schedule planning, environmental-control discussion	3 h	Individual sleep/fatigue plan and monitoring example
S2	Emotional stress self-regulation	Guided skills practice, confidential self-management planning	3 h	Use of an agreed self-regulation strategy in a low-stakes scenario
S3	Crew communication, peer support, and conflict management	Facilitated team exercise, role-play, structured debriefing	3 h	Closed-loop communication and concern-escalation rehearsal
U1	Diving physiology, equipment, and risk recognition	Brief teaching, equipment laboratory, and pre-dive check practice for already qualified/authorized external-work personnel	4 h	Completed locally approved pre-dive and buddy check
U2	Underwater operations and emergency drills	Confined-water or mission-rehearsal drills for already qualified/authorized external-work personnel, with structured debriefing	7 h	Observed procedural sequence and team debriefing record
Total	Curriculum	Mixed instructional program	40 h	Illustrative allocation; not a validated dose or certification requirement

Assessment was deliberately separated into two levels. Training programs may collect formative evidence, such as a locally approved skills checklist, structured handoff, or scenario debrief, to guide feedback and remediation. Accredited or local standards may define whether a practice attempt is satisfactory for that setting. The present paper supplies no universal passing score, critical-error threshold, or deployment rule. Any operational readiness decision requires separate governance, clinical oversight, and prospective validation.

The curriculum is intended to be adaptable at two levels. Core elements are the risk-capability logic, the module boundaries, and the observable outcomes. Adaptable elements include teaching materials, equipment-specific drills, language, sequence, duration, provider qualifications, environmental controls, and local escalation contacts. A program that changes an adaptable element should document the change and its reason. A program that changes a core element should state how the relevant risk-action link is preserved.

### Ethics and transparency

No participants were enrolled at any stage. The exercise used no identifiable personal data and collected no participant responses. On that basis the Institutional Review Board of Soonchunhyang University Cheonan Hospital granted an exemption from review (IRB No. 2026-01-015).

The standardized scenario, analytic rules, domain architecture, module descriptions, and 28 learning outcomes are reported in full. Readers can therefore inspect and challenge the assumptions. An outcome-level evidence-to-rationale audit is provided in Supplementary Table S1.

## Results

### Scenario-derived risk architecture

The scenario concentrated attention on occupational and operational hazards that arise while a small non-medical crew lives and works inside a constrained, pressurized environment. Table 2 defines a planned 15-m, 14-day design case; it is no protocol for deeper saturation exposure, commercial diving, or an autonomous medical unit. The boundary is narrow by design. Evidence mapping produced eight recurrent risk-capability clusters: emergency recognition and initial stabilization; remote medical communication and structured handoff; evacuation linkage and medical-kit use; musculoskeletal load and fatigue; nutrition, hygiene, and self-monitoring; emotional stress and self-management; sleep, team support, and conflict; and diving safety with operational control. These clusters were iteratively resolved into the final four-domain, ten-module curriculum architecture organized around occupational health and safety. Figure 1 shows the complete pathway, and Table 3 lists all 28 outcomes.

### Domain and module architecture

The first domain, emergency preparedness, holds three modules: E1 Initial response to life-threatening events, E2 Structured medical communication and telemedicine, and E3 Underwater-specific emergencies and evacuation linkage. E1 concentrates on recognition, first response, and role limits. E2 addresses the quality of information transmitted to a topside clinician or emergency service. E3 links pressure-related illness, fire, smoke, carbon dioxide concern, trauma, and evacuation preparation to local scenario drills.

The second domain, physical self-maintenance, holds P1 Musculoskeletal load and fatigue management and P2 Nutrition, hydration, hygiene, medication, and self-checks. Both modules are framed as routine preventive work practices. Neither prescribes a universal exercise or dietary regimen. Local clinicians and operational leads would adapt movement instruction, medication procedures, and infection-control detail to the habitat, the crew, the equipment, and the occupational regulations in force.

The third domain, psychological and team functioning, is deliberately divided into S1 Sleep and circadian management, S2 Emotional stress self-regulation, and S3 Crew communication, peer support, and conflict management. The division addresses a conceptual problem that arises when sleep, personal emotion regulation, and interpersonal conflict are treated as one competency. The three concerns are clearly related. They nonetheless demand different mechanisms, teaching methods, privacy protections, and performance observations.

The fourth domain, underwater operational safety, comprises U1 Diving physiology, equipment, and risk recognition and U2 Underwater operations and emergency drills. Its purpose is to organize safety behaviors relevant to the defined mission. U1 and U2 supplement existing qualification. They confer no professional diving competence. External-task performance outcomes apply only to personnel already qualified, medically cleared, and authorized under local rules; other crew members receive only the role-relevant orientation required by the operation.

The architecture uses ten modules. The count itself carries no significance. Module boundaries were chosen because they support a practical teaching decision: who should teach the content, what kind of practice is required, what privacy or clinical governance applies, and what evidence might show that the content was encountered. A single instructor may deliver more than one module where appropriately qualified, though a shared teacher does not make the learning functions identical.

Every module carries a route back to the standardized scenario. Emergency modules refer to delayed definitive care and a limited local role. Physical modules attach to work pacing and routine living. Psychological and team modules address small-crew functioning and confidentiality. Operational modules cover task preparation and supervised response. The architecture is a curriculum map. It ranks neither personal worth nor numerical risk.

### Illustrative delivery plan

Table 4 gives one hypothetical 40-h implementation example: 12 h for emergency preparedness, 8 for physical self-maintenance, 9 for psychological and team functioning, and 11 for underwater operational safety. The hours are illustrative only. They are not core curriculum elements, recommended minimums, certification requirements, or a validated dose. The example simply assigns more time to content requiring supervised demonstration, rehearsal, or integrated drills. Programs may need additional time for accredited BLS/AED training, equipment orientation, medical simulation, or a supervised mission rehearsal.

Delivery is intentionally mixed across methods. Knowledge-intensive material can move through short teaching units, digital material, and guided discussion. Skills bearing on immediate safety require supervised practice with feedback. Team and communication components require scenario work and structured debriefing. The distinction follows the proposed learning mechanism, and it sets aside any assumption that one method can carry all content.

A practical implementation could run in three blocks. Block 1 establishes the scenario, individual role boundaries, basic emergency recognition, and routine self-management. Block 2 develops structured communication, equipment checks, workload pacing, sleep planning, emotional self-regulation, and crew interaction. Block 3 runs integrated scenarios in which apparently separate issues arrive together: fatigue during a maintenance task, a communication failure during a medical concern, or a sleep-deprived crew member facing an escalating environmental problem. The blocks are illustrative, and they should never bypass locally required credentialing.

Facilitation requires a multidisciplinary arrangement. Emergency-response material should be delivered or overseen by instructors qualified for the applicable BLS, AED, and first-aid curriculum. Diving and equipment components require instructors authorized within the relevant operational system. Psychological and team-functioning components require confidentiality safeguards and explicit limits on peer support. Occupational health input links workload, fatigue, ergonomic prevention, preventive self-management, and confidential health escalation to the actual task environment. It also connects these worker-level controls to local work organization and surveillance pathways. Role ownership stays visible.

### Implementation-oriented assessment blueprint

Formative evidence of learning and any later readiness decision are held apart in Table 5. Module-specific questions can document knowledge acquisition; task checklists can structure practice; a Situation-Background-Assessment-Recommendation (SBAR) handoff can make communication visible; and integrated scenarios can support team feedback. Local approved criteria define satisfactory performance during practice. None of these observations has established predictive validity for mission outcome. That question requires prospective testing.

**Table 5:** Formative assessment and separate optional health-support blueprint.

Assessment dimension	Potential evidence or tool	Intended use	Important limit
Knowledge	Module-specific questions using locally reviewed content	Identify areas requiring further teaching	No universal passing score is proposed.
Emergency skills	Instructor observation checklist aligned with the approved BLS/AED and first-response curriculum	Structure feedback and repeated practice	Checklists require local instructor training and do not establish field validity.
Communication	SBAR-style handoff exercise, vital-sign transmission review, telemedicine contact rehearsal	Make information quality and escalation visible	Must match the actual emergency and telemedical pathway.
Team performance	Integrated scenario, structured debrief, role/communication observations	Support learning about coordination and adaptation	Simulation performance is not yet a validated predictor of mission outcome.
Physical self-management	Task pacing plan, self-check record, fatigue discussion	Support routine prevention and early reporting	Requires local ergonomic and medical adaptation.
Optional occupational health/clinical support	If locally adopted, PSS, PHQ-9, GAD-7, or ISI under a prespecified clinical or occupational health protocol (34–41)	Identify a need for confidential clinical review or support outside curriculum scoring	Not required by the curriculum. Access is limited to designated clinical personnel; participation status, data handling, and escalation must be specified locally. Elevated results do not trigger automatic exclusion.
Operational decision	Independent organizational process combining regulations, medical oversight, training records, and mission-specific controls	Determine whether a person may be assigned	This manuscript provides no deployment algorithm or threshold.

Table 5 also separates optional occupational health or clinical support from curriculum assessment. The curriculum does not require screening instruments. If PSS, PHQ-9, GAD-7, or ISI measures are used (34–41), they should be administered only under a prespecified clinical or occupational health process and never as peer instructor curriculum scores. Access should be limited to designated clinical personnel. Local governance must specify whether use is voluntary or mandatory, how results are retained or disclosed, and which escalation route applies. An elevated result should trigger confidential clinical review or referral according to local policy, not automatic deployment exclusion. These measures form no composite readiness score.

## Discussion

This paper provides a preliminary occupational health curriculum for pre-deployment preparation in a bounded underwater-habitat workplace. Its practical contribution is inspectable development logic: a bounded scenario yields occupational and operational risk-action statements, those statements yield separable worker capabilities, and the capabilities yield modules and observable learning outcomes. Figure 1 displays that pathway. Worker protection is the organizing purpose. Validation remains a later stage.

The scenario focus carries weight. Underwater habitats share elements with saturation diving, scientific diving, submarines, and ICE analogs, yet the settings are not interchangeable. Pressure exposure, limited access to care, and external underwater work make diving medicine and safety standards directly relevant (6–9, 21, 25, 26). Isolation, sleep disruption, small-team dependence, and constrained privacy explain the relevance of spaceflight and submarine analog evidence (4, 5, 10–16, 27, 28). Each source family is used for the problem it illuminates, and no single environment is treated as a complete proxy for another.

The emergency domain is deliberately limited to a non-medical first-response role. A crew member should be able to identify a concerning event, perform trained basic actions, deliver a structured account, and prepare for escalation. Advanced invasive procedures and independent diagnosis stay outside the curriculum. This scope sits comfortably with resuscitation guidance, telemedicine implementation principles, prehospital decompression-illness care, and the practical constraints described in confined-space rescue work (17–23).

Physical self-maintenance is treated as an operational capacity. Inside a constrained habitat, exercise, pacing, hydration, food choices, skin care, and routine self-checks all bear on sustained function, and their value is operational rather than a matter of personal wellness alone. Spaceflight research supports the relevance of resistance exercise and nutritional countermeasures in prolonged missions (42, 43). General activity guidance and sleep recommendations offer a starting point for adaptation, while light-based and behavioral interventions suggest options to be tailored to the environment and to individual needs (44–48).

Separating the psychological content is central to this specification. Sleep and circadian planning, emotional self-regulation, and team interaction overlap, and folding them into one competency obscures both content and governance. Sleep management can be taught through planning, environmental adjustment, and monitoring. Emotional self-regulation may involve stress recognition, breathing, relaxation, or cognitive strategies. Crew communication and conflict management call for role clarity, check-ins, escalation rules, and safeguards around privacy. Evidence for mindfulness, digital stress interventions, and space-oriented psychological tools supports consideration of these strategies, though none of it establishes that they will prevent adverse outcomes in a specific underwater habitat (49–54).

The division also clarifies what should follow when a concern arises. A sleep record may open a discussion about work scheduling or fatigue controls. A stress-management exercise may help a participant interrupt acute arousal during a drill. A conflict-management process may require a facilitated conversation about roles or decision authority. A serious psychological concern should activate a confidential clinical or supervisory pathway. The four situations differ in kind. Treating them as a single peer-support issue would blur responsibility and could create avoidable privacy risk.

The module structure is compatible with competency-based education if the term is used carefully. Competency is demanding. That distinction is important for interpretation. Attendance, online completion, or a favorable instructor impression cannot establish it. A defensible program needs stated behavior, a relevant context, trained assessors, feedback, and an observation method appropriate to the decision. The present framework supplies observable outcomes and performance contexts, while Table 4 provides candidate formative evidence. Accredited local standards may define satisfactory BLS, equipment-check, or drill performance; framework-specific readiness thresholds require future validation.

The assessment blueprint is intentionally conservative. Formative observations remain educational evidence, while optional clinical or occupational health tools sit outside curriculum scoring. This separation matters. Converting either source into a single clearance score would require prospective evidence, independent oversight, and an explicit governance process.

Readiness decisions in extreme environments usually combine medical fitness, statutory qualification, operational authorization, situational risk, and organizational responsibility. A brief educational scoring system cannot replace those elements. A training framework contributes one source of information: whether specified content was delivered, and whether a learner had opportunities to demonstrate defined behavior under supervision. It cannot decide alone that a person is safe to deploy. Keeping that distinction explicit is a central safeguard in the proposed implementation model.

The occupational health perspective serves as the organizing framework connecting routine prevention, acute response, communication, physical self-management, psychosocial functioning, and task-specific safety. Occupational health services can coordinate workload and ergonomic prevention, fatigue management, routine health support, early reporting, and confidential clinical escalation. Diving operations retain authority for diver qualification, equipment, dive procedures, and technical rescue. Emergency and clinical services retain responsibility for accredited response training, telemedical pathways, and clinical decisions, while mission management retains assignment and deployment authority. These role boundaries matter in practice. Whether the curriculum reduces injury, illness, evacuation, or mission failure remains unestablished.

Implementation requires governance as much as teaching. An operational program should identify who owns the emergency plan, who may revise training material, who handles a confidential concern, where clinical decisions sit, and how incident learning returns to the curriculum. These responsibilities do not belong to the trainee. Remote support requires the same clarity: a communication exercise is meaningful only when a real contact pathway, working equipment, and local decision authority have been confirmed.

The curriculum also draws a line between surveillance and punishment. A self-check, fatigue report, sleep record, or psychological concern should carry a stated purpose and a confidential handling process. If trainees expect ordinary reporting to trigger automatic exclusion, early reporting will be suppressed. If reporting draws no defined response, it becomes a formality. A future implementation study should examine acceptability, trust, reporting burden, and the consequences of escalation, alongside test scores.

Resource requirements deserve a place in any pilot record. They include instructor time, access to a simulation setting, availability of approved equipment, communication infrastructure, clinical backup, interpreter or language needs, participant time away from mission preparation, and the burden of repeated drills. A curriculum that is conceptually coherent yet undeliverable in the planned environment will not solve the operational problem. Feasibility therefore covers logistics, governance, workload, and acceptability as well as educational content.

Several research designs could advance the evidence base. A prospective before-and-after pilot could evaluate instructor usability, learner understanding, and change in observed drill behavior. A multi-site implementation study could compare context, adaptations, fidelity, and resource requirements. An analog-habitat study could track how sleep, workload, communication, and scenario performance unfold over time. No single design proves that a framework prevents serious events. Together they can clarify feasibility, mechanisms, and the limits of generalization.

### Strengths and limitations

One strength is the explicit boundary drawn between framework development and later validation. The development trail is open to inspection. The scenario, evidence roles, analytic rules, module boundaries, 28 outcomes, and candidate formative assessments are reported, and the revised supplements provide reproducible search syntax and a complete retained-source inventory. The framework also resolves conceptual overlap among sleep, stress, and crew conflict.

Several limitations remain. Evidence mapping was purposive, no formal quality appraisal was performed, independent duplicate screening was not undertaken, and no prespecified saturation rule was used. Development remained internal to the author group. No independent content review, learner or operator co-design, instructor usability test, participant assessment, pilot training, analog-habitat observation, or outcome comparison was undertaken. The curriculum is tied to a 15-m, 14-day, non-medical crew design case and may be unsuitable for deeper, longer, medically staffed, or differently regulated operations. It is a preliminary specification.

The reference base spans evidence with different purposes and degrees of directness. Operational and clinical guidance supports required procedures and role boundaries; direct underwater or diving evidence informs setting-specific hazards; submarine, ICE, and spaceflight studies provide adjacent analogue evidence; and general behavioral studies inform transferable self-management strategies. Supplementary Tables S1, S3 show these distinctions. The mixture is deliberate. It should not be mistaken for one body of direct efficacy evidence.

Future work should proceed in stages. Independent review should first involve prospective trainees, instructors, habitat operators, diving supervisors, telemedicine providers, occupational health clinicians, diving medicine experts, emergency clinicians, and instructional designers. Structured stakeholder review or cognitive interviewing can test clarity, feasibility, role boundaries, and content relevance. Instructors should then test materials, timing, and scenario logistics. A prospective analog pilot can evaluate acceptability, knowledge and skill change, communication quality, and simulation performance. Only later should proposed indicators be tested against operational outcomes or readiness decisions.

## Conclusion

An occupational health curriculum for an underwater habitat should state what is taught, why it is taught, and what remains unknown. The scope here is deliberately narrow. Four domains, ten modules, and 28 observable outcomes define a preliminary occupational health curriculum for the pre-deployment preparation of non-medical aquanaut candidates in the 15-m, 14-day design case.

The specification is evidence-informed and unvalidated. It provides a transparent starting point for implementing an occupational health curriculum, independent stakeholder review, instructor testing, and prospective pilot evaluation while remaining subordinate to diving qualification, clinical governance, and mission authority. It does not establish mission readiness, deployment eligibility, or safety effectiveness.

## Data Availability

The original contributions presented in the study are included in the article/Supplementary material, further inquiries can be directed to the corresponding author.
