# Supplementary material for: An evidence-informed occupational health curriculum for pre-deployment preparation of aquanauts in underwater habitats: a scenario-based framework development study
Source: Front Public Health. 2026 Sep 25;14:1962851. doi: 10.3389/fpubh.2026.1962851 (PMC13649977; doi:10.3389/fpubh.2026.1962851)
Supplement: Supplementary file 1 [file Supplementary_file_1.docx]

**Supplementary material**

Supplementary Table S1. Outcome-level evidence-to-rationale traceability audit. Evidence-role codes: O, authoritative operational/clinical guidance; D, direct underwater/diving evidence; A, adjacent analogue evidence; G, general behavioral/health evidence.

| **Outcome** | **Evidence anchor** | **Risk/action rationale and boundary** |
| --- | --- | --- |
| E1-1 | [O] Resuscitation and education guidance (18–20) | Predefined red flags trigger trained response and escalation; the outcome does not require diagnostic labeling. |
| E1-2 | [O] Resuscitation guidance (18–20) | Basic response skills are teachable through an accredited, locally approved curriculum; the outcome excludes advanced procedures. |
| E1-3 | [O] Emergency-system guidance (18–20); confined-space rescue (23) | Role boundary, scene safety, and activation of support limit unsafe role expansion. |
| E2-1 | [O+D] WHO telemedicine guidance (17); diving telemedicine (22) | A structured account supports remote decision-making when direct assessment is constrained. |
| E2-2 | [O] WHO telemedicine guidance (17); emergency communication principles (18–20) | Time, units, trends, and operational context affect the usefulness of transmitted observations. |
| E2-3 | [O+D] WHO telemedicine guidance (17); diving telemedicine (22) | The content is a pathway rehearsal; contact numbers, kit contents, and authority remain local. |
| E3-1 | [D] Diving medicine and decompression guidance (8, 9, 21) | Pressure-related and habitat hazards require recognition of warning signs or exposure histories and safe first actions, without diagnostic differentiation. |
| E3-2 | [D] Prehospital decompression guidance (21); confined-space rescue (23) | Initial protection and preparation can be taught without assigning independent clinical or rescue authority. |
| E3-3 | [D] Diving telemedicine (22); confined-space rescue (23) | Evacuation linkage requires an agreed handoff and transport pathway; the destination is site specific. |
| P1-1 | [D+A] Aquatic and extreme-environment physiology (6, 7); resistance-exercise and nutrition countermeasures (42, 43) | Movement preparation and handling technique address task-related strain; exercise prescription must be locally adapted. |
| P1-2 | [A+G] Extreme-environment physiology (6, 7); general physical activity and sedentary-behavior guidance (44) | Fatigue indicators may affect safe pacing and attention before a task becomes an emergency. |
| P1-3 | [A+G] Physical activity guidance (44); spaceflight countermeasure evidence (42, 43) | Pacing and stop/report actions are operational controls, not a universal fitness standard. |
| P2-1 | [A+G] Long-duration nutrition evidence (42); physical activity guidance (44) | Hydration, food, permitted medication, hygiene, and self-check routines support routine health maintenance. |
| P2-2 | [D] Habitat scenario; aquatic/extreme physiology (6, 7) | The broad preventive content requires local infection-control, medication, and wound-care procedures. |
| P2-3 | [O] Telemedicine implementation guidance (17) | A new symptom has value only when a defined reporting and response pathway exists. |
| S1-1 | [A+G] ICE evidence (4, 5); spaceflight sleep evidence (13); sleep guidance (45–48) | Schedule and environment can disrupt sleep; the outcome focuses on recognizing modifiable contributors. |
| S1-2 | [G] Sleep guidance and intervention evidence (45–48) | Planning protects sleep opportunity and fatigue management without prescribing a universal sleep intervention. |
| S1-3 | [A] ICE/spaceflight sleep evidence (4, 5, 13) | Monitoring is intended to prompt discussion or support, not covert surveillance or automatic exclusion. |
| S2-1 | [A+G] Psychological countermeasure literature (14–16); stress intervention evidence (49–52) | Recognition of arousal supports timely use of an agreed coping strategy. |
| S2-2 | [G] Mindfulness and digital stress-management evidence (49–52) | The outcome specifies practice of low-risk self-regulation rather than clinical psychotherapy. |
| S2-3 | [A] Space-oriented psychological support literature (53, 54) | Confidential help-seeking needs an escalation process that is separate from routine peer support. |
| S3-1 | [D+A] Team and crew-functioning research (10–12); saturation/submarine evidence (24, 27, 28) | Routine check-ins and closed-loop communication support role clarity and shared situation awareness. |
| S3-2 | [A] Team-dynamics evidence (10–12); ICE evidence (4, 5) | A structured concern-and-resolution process prevents conflict content from being hidden inside a sleep or mood module. |
| S3-3 | [A] Crew mental-health and submarine evidence (16, 27, 28) | Peer support has limits; clinical or supervisory escalation must be explicitly available. |
| U1-1 | [D+O] Aquatic/pressure physiology (6–9); diving standards (25, 26) | A role-appropriate explanation of risk informs safer decisions without conferring professional diving authority. |
| U1-2 | [O] NOAA and U.S. Navy diving standards (25, 26) | Pre-dive and buddy checks are operational controls that must match local equipment and regulations. |
| U2-1 | [O] Diving standards (25, 26) | Entry/exit, communication, and task-abort signals require supervised rehearsal in the actual or analogous setting. |
| U2-2 | [O+D] Diving standards and confined-space rescue evidence (23, 25, 26) | Scenario drills support coordination during loss of control; they do not validate field rescue performance. |

Supplementary Table S2. Reproducibility aid for evidence identification and source selection

| Source / concept block | Search date | Syntax or repository query terms | Selection logic |
| --- | --- | --- | --- |
| PubMed: underwater/diving hazards | Last update: 4 May 2026 | ("underwater habitat"[Title/Abstract] OR aquanaut*[Title/Abstract] OR "saturation diving"[Title/Abstract] OR "saturation diver"[Title/Abstract]) AND (physiology[Title/Abstract] OR "decompression illness"[Title/Abstract] OR barotrauma[Title/Abstract] OR "oxygen toxicity"[Title/Abstract] OR "nitrogen narcosis"[Title/Abstract] OR "carbon dioxide"[Title/Abstract]) | Retain sources describing scenario-relevant hazards, mechanisms, recognition cues, first actions, or escalation. |
| PubMed: emergency/telemedicine | Last update: 4 May 2026 | ("underwater habitat"[Title/Abstract] OR diving[Title/Abstract] OR submarine*[Title/Abstract] OR "confined space"[Title/Abstract]) AND (telemedicine[Title/Abstract] OR emergency[Title/Abstract] OR resuscitation[Title/Abstract] OR "first aid"[Title/Abstract] OR evacuation[Title/Abstract] OR handoff[Title/Abstract]) | Retain sources informing non-medical recognition, trained first response, structured communication, or evacuation linkage. |
| PubMed: ICE/team/sleep | Last update: 4 May 2026 | ("isolated and confined"[Title/Abstract] OR "isolated confined extreme"[Title/Abstract] OR spaceflight[Title/Abstract] OR submarine*[Title/Abstract]) AND (sleep[Title/Abstract] OR circadian[Title/Abstract] OR stress[Title/Abstract] OR "mental health"[Title/Abstract] OR teamwork[Title/Abstract] OR communication[Title/Abstract] OR conflict[Title/Abstract]) | Use as adjacent analogue evidence for transferable sleep, psychological, and team-functioning concepts; do not derive diving procedures from analogue evidence alone. |
| PubMed: physical self-maintenance | Last update: 4 May 2026 | (spaceflight[Title/Abstract] OR "extreme environment"[Title/Abstract] OR aquatic[Title/Abstract]) AND (fatigue[Title/Abstract] OR exercise[Title/Abstract] OR nutrition[Title/Abstract] OR hydration[Title/Abstract] OR "physical activity"[Title/Abstract]) | Retain sources informing fatigue recognition, pacing, movement, nutrition, hydration, or self-maintenance that can be locally adapted. |
| WHO public repository | Accessed through 4 May 2026 | telemedicine implementation guide; remote care; emergency communication | Prioritize official implementation guidance for telemedical pathways, information transfer, and governance. |
| NOAA public repository | Accessed through 4 May 2026 | NOAA diving standards and safety manual; scientific diving safety; buddy check; emergency procedure | Use authoritative operational guidance for diving safety behaviors; site-specific rules remain controlling. |
| U.S. Navy public repository | Accessed through 4 May 2026 | U.S. Navy diving manual; diving emergency; decompression; equipment check | Use authoritative operational guidance for pressure-related risk and diving procedures; do not generalize equipment-specific details. |
| NASA public repository | Accessed through 4 May 2026 | NEEMO underwater habitat; isolated confined environment; astronaut sleep; psychological countermeasures; team performance | Use habitat and spaceflight analogues for context, sleep, stress, and team-functioning concepts only. |

Note: The search was purposive rather than systematic. The PubMed strings above operationalize the concept blocks reported in the original development process and are provided to permit reproducible re-searching; no retrospective screening count or formal saturation estimate is claimed.

Supplementary Table S3. Complete retained evidence-source inventory and evidence role

| Ref. | Source shorthand | Evidence role / directness | Primary curricular use |
| --- | --- | --- | --- |
| 1 | Lee et al., 2026 | D | Aquanaut selection context; distinguishes pre-selection evaluation from pre-deployment education. |
| 2 | Koutnik et al., 2021 | D | Multiday saturation/NEEMO physiology and habitat context. |
| 3 | Todd & Reagan, 2003 | D/A | NEEMO habitat mission structure and analog operations. |
| 4 | Pagel & Choukèr, 2016 | A | Isolation and confinement effects relevant to small-crew functioning. |
| 5 | Palinkas & Suedfeld, 2021 | A | ICE psychosocial risks, privacy, adaptation, and team dependence. |
| 6 | Pendergast et al., 2015 | D | Aquatic physiology and immersion-related mechanisms. |
| 7 | Grocott, 2008 | A | Extreme-environment physiology and functional risk context. |
| 8 | Mitchell, 2024 | D | Decompression-illness recognition and clinical context. |
| 9 | Moon & Mitchell, 2019 | D | Decompression-sickness treatment context and escalation need. |
| 10 | Bell et al., 2019 | A | Team dynamics in long-duration analog missions. |
| 11 | Blackwell Landon et al., 2017 | A | Team-performance considerations for exploration crews. |
| 12 | Landon et al., 2018 | A | Teamwork and collaboration in extreme missions. |
| 13 | Barger et al., 2014 | A | Spaceflight sleep deficiency and circadian risk. |
| 14 | Manzey & Schiewe, 1992 | A | Psychological training strategies in astronaut preparation. |
| 15 | Manzey et al., 1995 | A | Psychological countermeasures for extended missions. |
| 16 | Oluwafemi et al., 2021 | A | Astronaut mental-health risks and support options. |
| 17 | WHO, 2022 | O | Telemedicine implementation, information transfer, and governance. |
| 18 | Merchant et al., 2020 | O | Resuscitation priorities and emergency response principles. |
| 19 | Song et al., 2021 | O | Korean adult BLS procedures. |
| 20 | Lee et al., 2021 | O | Resuscitation education and system implementation. |
| 21 | Mitchell et al., 2018 | D | Prehospital decompression-illness first actions and escalation. |
| 22 | Wölfel et al., 2011 | D | Diving-accident telemedicine and symptom communication. |
| 23 | Petinaux et al., 2014 | D/A | Confined-space rescue constraints and casualty movement. |
| 24 | Romsbotn et al., 2022 | D | Psychosocial job demands among saturation divers. |
| 25 | NOAA, 2023 | O | Scientific-diving checks, buddy systems, equipment control, and emergencies. |
| 26 | U.S. Navy, 2016 | O | Diving procedures, pressure-related risk, and emergency operations. |
| 27 | Hughes et al., 2024 | A | Submarine medical evacuation and mission-continuity context. |
| 28 | Rapley et al., 2017 | A | Submarine psychological support and hygiene. |
| 29 | Skivington et al., 2021 | Methodological | Complex-intervention development, context, and staged evaluation. |
| 30 | O’Cathain et al., 2019 | Methodological | Development guidance for complex health interventions. |
| 31 | Gale et al., 2013 | Methodological | Framework-analysis traceability principles. |
| 32 | Hoffmann et al., 2014 | Methodological | TIDieR reporting logic for replicable intervention description. |
| 33 | Arksey & O’Malley, 2005 | Methodological | Scoping-method rationale for mapping an emerging field. |
| 34 | Cohen et al., 1983 | G / optional clinical support | Original PSS instrument; not a curriculum outcome measure. |
| 35 | Kroenke et al., 2001 | G / optional clinical support | Original PHQ-9 instrument; not a curriculum outcome measure. |
| 36 | Spitzer et al., 2006 | G / optional clinical support | Original GAD-7 instrument; not a curriculum outcome measure. |
| 37 | Bastien et al., 2001 | G / optional clinical support | Original ISI validation; not a curriculum outcome measure. |
| 38 | Lee et al., 2015 | G / optional clinical support | Korean PSS validation. |
| 39 | Han et al., 2008 | G / optional clinical support | Korean PHQ-9 validation. |
| 40 | Ahn et al., 2019 | G / optional clinical support | Korean GAD-7 validation. |
| 41 | Cho et al., 2014 | G / optional clinical support | Korean ISI validation. |
| 42 | Smith et al., 2005 | A | Long-duration spaceflight nutrition status. |
| 43 | Smith et al., 2012 | A | Resistance exercise and nutrition countermeasures. |
| 44 | Bull et al., 2020 | G/O | General physical-activity guidance for locally adapted prevention. |
| 45 | Hirshkowitz et al., 2015 | G | General sleep-duration guidance. |
| 46 | van Maanen et al., 2016 | G | Light-based sleep intervention evidence. |
| 47 | Trauer et al., 2015 | G | CBT-I evidence informing sleep-management options. |
| 48 | Zachariae et al., 2016 | G | Digital CBT-I evidence informing adaptable sleep strategies. |
| 49 | Goyal et al., 2014 | G | Meditation/stress-management evidence. |
| 50 | Khoury et al., 2015 | G | Mindfulness-based stress reduction evidence. |
| 51 | Heber et al., 2017 | G | Web/computer stress-intervention evidence. |
| 52 | Ebert et al., 2016 | G | Employee digital stress-management intervention evidence. |
| 53 | Pagnini, 2024 | A | Space-oriented psychological-support tools. |
| 54 | Smith, 2024 | A | Automated psychotherapy considerations for spaceflight; contextual only. |

Evidence-role codes used in Supplementary Table S1: O = authoritative operational/clinical guidance; D = direct underwater/diving evidence; A = adjacent analogue evidence; G = general behavioral/health evidence. Methodological references support framework development or reporting rather than a specific learning outcome.
